# Processing Matters in Nutrient-Matched Laboratory Diets for Mice—Energy and Nutrient Digestibility

**DOI:** 10.3390/ani11020523

**Published:** 2021-02-17

**Authors:** Linda F. Böswald, Jasmin Wenderlein, Reinhard K. Straubinger, Sebastian Ulrich, Ellen Kienzle

**Affiliations:** 1 Chair for Animal Nutrition and Dietetics, Ludwig-Maximilians-Universität München, 85764 Oberschleißheim, Germany; kienzle@tiph.vetmed.uni-muenchen.de; 2 Institute for Infectious Diseases, Ludwig-Maximilians-Universität München, 80539 Munich, Germany; jasmin.wenderlein@micro.vetmed.uni-muenchen.de (J.W.); Reinhard.Straubinger@micro.vetmed.uni-muenchen.de (R.K.S.); ulrich@micro.vetmed.uni-muenchen.de (S.U.)

**Keywords:** standardization, carbohydrate digestibility, feed processing, starch gelatinization, gut

## Abstract

**Simple Summary:**

The processing of feed items has an impact on their nutritive properties, e.g., differences in the degree of starch gelatinization between pelleted and extruded diets are apparent. In many species, including humans, it is known that this influences the digestion process on enzymatic and microbial levels, and thus the animals´ ability to utilize the diet. Laboratory animal diets are often marketed as identical products, e.g., a standard maintenance diet, which can be purchased in pelleted or extruded form. The hypothesis that there are differences in energy and nutrient digestibility among such products, even though they are claimed to be the same diet, was investigated. The results of the digestibility trials confirm the hypothesis. Additionally, they show that even among batches of the same laboratory rodent diet in the same form, standardization is not always achieved.

**Abstract:**

Starch gelatinization is a major determinant of carbohydrate digestibility and varies with diet processing. Laboratory rodent diets are often marketed as identical, but are sold in different forms, regardless of the markedly higher starch gelatinization in extruded than in pelleted diets. Our hypothesis was that this would impact energy and nutrient digestibility in mice fed pellets or extrudate, respectively. Trial 1 showed that feeding C57BL/6 mice a standard maintenance diet in extruded form results in a significantly higher digestibility of organic matter, energy, and carbohydrates than the identical diet in pelleted form. The replication of the experiment, however, revealed a variation between batches of the same pelleted diet regarding starch and total dietary fiber contents. Given the significant differences in diet digestibility and the potential impacts of digestibility on nutrient utilization, the intestinal microbiome, and intermediary metabolism, trials performed with differently processed diets are not comparable. This might partly explain failures to reproduce results, especially in gastrointestinal or microbiome research. Considering this impact on experimental animals, the degree of starch gelatinization should be declared in the diet information for laboratory animal diets. The differences between batches of laboratory animal diets as observed in the pellets are not acceptable.

## 1. Introduction

Laboratory animal diets are available in different forms, e.g., pelleted, extruded, and paste-like forms, or in the form of a meal or powder. Each form has its own benefits and disadvantages, however pellets are used most often. Pelleted and powdered variations of the same diet resulted in significant differences in growth and body composition in C57BL/6 mice [1]. Levy et al. [2] investigated the effects of pelleted vs. extruded diets in two strains of laboratory mice. They found significant effects of the diet form and strain of mice on feed disappearance, mass of excreta, and cage soilage. In terms of animal nutrition, the relation between feed intake and fecal mass can give a rough estimate of feed utilization. Taking into account the differences in the processing techniques for pelleting and extrusion, the properties of dietary carbohydrates, i.e., starch, may explain some of the effects described by Levy et al. [2]. Starch is the main carbohydrate source in typical laboratory mouse diets; its properties are highly variable depending on source and processing type and impact the nutritive effects [3].

When treated with heat and moisture, starch will gelatinize [4]. The starch granules from plant materials are destroyed to a certain degree and the altered starch molecules become partly soluble, and thus more degradable, primarily by amylase, in the gastrointestinal tract. This can be quantified by the degree of starch gelatinization and varies with the type of processing a diet is subjected to. Extrusion uses the combination of shearing forces, heat, moisture, and pressure to partly destroy the granules [5]. Pelleting is a low-moisture, thermo-mechanical method of processing [6] that does not generate as much shear forces or heat as extrusion does [7].

In farm animal nutrition, the total starch content and degree of starch gelatinization are important parameters in assessing the effect on animal performance [4]. Previous observations have shown that the degree of starch gelatinization varies between the differently processed types of supposedly identical laboratory animal maintenance diets, i.e., pellets vs. extrudate [8].

Gelatinized starch can be digested more easily via the pancreatic amylase and disaccharidases in the small intestine of monogastric mammals, resulting in the absorption of glucose. Starch escaping digestion in the small intestine passes on into the large intestine, where it may be used as a substrate for microbial fermentation or is excreted via feces. The metabolites of microbial fermentation, volatile fatty acids (VFAs) and lactate, contribute to enterocyte nutrition, and if absorbed, to the host organism´s energy supply [9,10]. A decrease in chyme pH indicates increased VFA synthesis, and therefore microbial fermentation due to higher availability of the substrate in various species [11,12,13,14,15]. However, glucose absorption from dietary starch is much higher in case of small intestinal enzymatic digestion, while in large intestinal fermentation more VFAs are absorbed.

In laboratory animal nutrition, maintenance diets are often marketed as pelleted and extruded products with identical product information. Even though it is known that the characterization of starch, especially in cereal-based products such as rodent diets, is important [16], labeled information on the degree of starch gelatinization is often lacking. A previous pilot study has shown that starch gelatinization differs significantly between such product pairs [8]. 

The aim of the present study was to show the impact of processing, i.e., pelleting vs. extrusion, on the growth and energy and nutrient digestibility of laboratory mice. Our hypothesis was that the higher degree of starch gelatinization in the extruded diet would lead to an overall higher energy digestibility as compared to apelleted diet.

## 2. Animals, Materials and Methods

### 2.1. Animals and Diets

Fifty-four young adult mice (C57BL/6 strain, female, purchased from envigo RMS B. V., Horst, Netherlands, at the age of 8 weeks) were used. They were housed in groups of 2–3 on silicate bedding (Tigerino Crystals, Matina GmbH, Munich, Germany) in isocages (Techniplast, Buguggiate, Italy) in a specific pathogen-free (SPF) facility. The mice were allocated to the experimental groups P (pelleted diet) and E (extruded diet) after a three-week adaptation period to the housing system. During the feeding trial, the mice were fed a commercial maintenance diet that is available either in pelleted (P) or extruded (E) form. Trials 1 and 2 were conducted following the same setup, but in two successive runs with different batches of the same diets to serve as a biological replication. The diets are described by the manufacturer to be based on soy and cereals (wheat, corn). Details of all diets are summarized in Table 1. 

Trial 1: Groups P and E consisted of 11 mice each. The P diet contained 27% starch, with a starch gelatinization degree of 22% (15% after autoclaving into the SPF facility). Diet E had the same starch content but higher degrees of starch gelatinization (64% before and 57% after autoclaving). Each group was distributed into five cages (4 × 2 mice and 1 × 3 mice per cage). 

Trial 2: Groups P and E consisted of 16 mice each (pair-housed in 8 cages per group). New batches of the same P and E diets as in trial 1 were purchased for trial 2 from the same manufacturer. Diet P had a starch content of 43%, with starch gelatinization degrees of 22% before and 17% after autoclaving. The starch content of diet E was 28%, with starch gelatinization degrees of 50% before and 70% after autoclaving.

### 2.2. Digestibility Trial

Body weight (BW) was recorded weekly. Daily feed intake per cage was documented by weighing the amount of diet offered and weighing the refusals after 24 h. After the first 27 days of the experiment, a balance trial with complete fecal collection was conducted for 14 days in trial 1. The results from this trial showed that a longer collection period is advisable to obtain sufficient fecal mass, so in trial 2 the collection period lasted 17 days. Feces were lyophilized, ground, and analyzed with standard methods (bomb calorimetry, Weende analysis) [17] to assess the following nutrients: dry matter (DM), gross energy (GE), crude protein (CP), crude ash (CA), HCl-insoluble ash, and ether extracts (EEThe diets were analyzed for DM, GE, CP, CA, HCl insoluble ash and EE contents using the same methods. The dietary starch content and gelatinization degree were analyzed following standard methods (VD LUFA III 7.2.6), while fecal starch content was determined using an enzymatic kit (Cat.-No. 10 207 748 035, Boehringer Mannheim, R-Biopharm, Darmstadt, Germany).

The apparent digestibility (aD) of GE, DM, and the nutrients was calculated as follows:

aD(nutrient) = (nutrient intake – fecal nutrient excretion)/nutrient intake * 100(1)

By subtraction of CA from DM, the organic matter (OM), and in turn aD(OM), could be calculated. The dietary and fecal contents of the carbohydrate and fiber fraction (CH + F) were calculated by subtracting CP, CA, and CE from DM. 

### 2.3. Organic Acids

Additional fecal samples were used for analysis of short-chain fatty acids and lactate. The samples were diluted 1:1 in distilled water and centrifuged (5000× *g* for 5 min). The supernatant was removed and laced with an internal standard and oxalic acid (2%), then centrifuged for 17 min (13,000× *g*). Gas chromatography was performed (Shimadzu GC 2010, Flame Ionization Detector, SGE BP21 capillary column). Concentrations in mmol/L were calculated according to a 6-point calibration procedure.

### 2.4. Post-Mortem Sampling

After 56 days on the respective diets, the mice were killed via cervical dislocation. The gastrointestinal tract (GIT) was removed (stomach and intestines) and weighed. In trial 2, the liver was also removed, weighed, and frozen (−18°C) for further analysis. The lyophilized livers of each pair of mice sharing a cage were ground and pooled for bomb calorimetry (GE determination, *n* = 8 per group). Chyme from the stomach, anterior and posterior small intestinal sites, cecum, and colon, as well as feces, was sampled, diluted with distilled water (1:5), and pH was measured (Inolab WTW pH Meter, Xylem Analytics Germany GmbH, Weilheim, Germany).

### 2.5. Statistics

The statistical analyses were conducted using SigmaPlot (Systat Software, San Jose, CA, USA). Comparisons between two diet groups from one trial were performed using a Student´s *t*-test (significance level set to *p* = 0.05). Calculations and statistics were conducted with data from individual animals, not cage units (*n* = 11 and *n* = 16 per group in trials 1 and 2, respectively). 

## 3. Results

### 3.1. Diets

The analyzed nutrient content did not completely match the labeled values. In four cases, the analyzed values lay outside the legal tolerance given in Regulation (EC) 767/2009, amended by Regulation (EC) 939/2010 (see Table 1). The nutrient contents also differed markedly between P1 and P2 and E1 and E2, respectively. 

Between the four diets used in the trials, there were differences in the degree of starch gelatinization. As expected based on the processing method, the extruded diets had a much higher degree of starch gelatinization than the pelleted diets. The degree of starch gelatinization was similar for P1 and P2, however the total starch contents differed considerably between these diets (27 vs. 43%, respectively, as fed), resulting in a difference in total intake of gelatinized starch in P1 vs. P2. Correspondingly, P2 had less total dietary fiber than the other diets.

### 3.2. Trial 1

**Figure 1:**
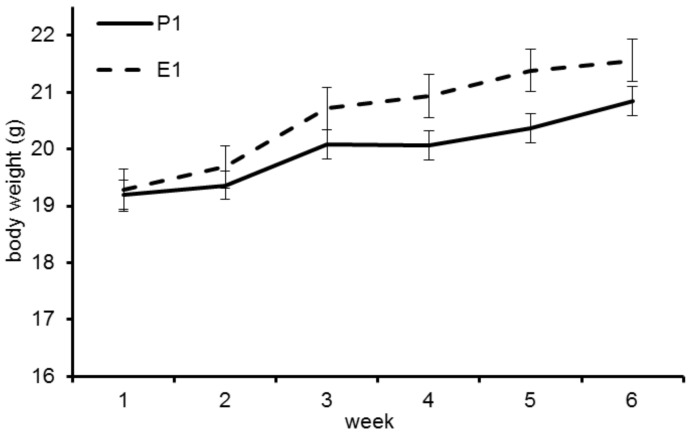
Body weight development of the mice in trial 1. Mice fed diet E1 show higher gains.

The initial BW did not differ significantly between groups P1 (19.36 ± 1.34 g) and E1 (19.69 ± 1.43 g; *p* = 0.61). BW increased in both groups during the trial, with higher BW in group E1 (Figure 1), however the difference in BW was not significant.

Mice maintained on diet E1 showed significantly higher apparent digestibility coefficients of GE, OM, and the CH+F (carbohydrate + fiber) fraction than group P1 (*p* < 0.001; Table 2). There were no significant differences in the digestibility of CP and EE between P1 and E1 (*p* = 0.31 and 0.09, respectively).

The pH levels of the colon chyme samples differed significantly between the groups (P1: 6.9 ± 0.3, E1: 7.2 ± 0.1, *p* < 0.05), but in the other sampling sites there were no significant differences (Table 3).

There was a trend of higher fecal acetic acid levels in P1 as compared to E1 (*p* = 0.09; Table 4). The ratio of acetic acid to propionic acid was markedly higher in P1 than E1 (7.15/1 vs. 5.92/1).

### 3.3. Trial 2

**Figure 2:**
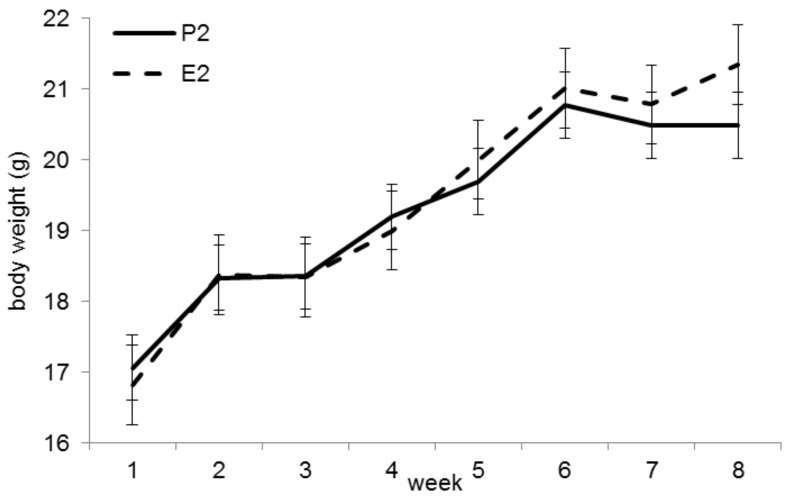
Bodyweight (BW) development in trial 2. The final BW (week 8) was significantly higher in group E2 (*p* < 0.05).

The initial BW did not differ between groups P2 and E2 (*p* = 0.53). At the end of the trial, animals in group E2 were significantly heavier (mean 21.3 ± 1.0 g vs. 20.5 ± 1.1 g, *p* < 0.05, Figure 2). Feed intakes were significantly higher in group E2 (4.31 ± 0.21 g/mouse/ day) than in group P2 (3.62 ± 0.33 g/mouse/day; *p* < 0.001).

Digestibility of GE, DM, and OM was significantly higher in diet P2 as compared to diet E2 (*p* < 0001; Table 2). For CP, digestibility was significantly higher in E2 (*p* < 0.001). The carbohydrate and fiber fraction showed significantly higher digestibility in P2 than E2 (*p* < 0.05). It has to be noted that group E2 showed highly similar digestibility coefficients as E1.

There were no significant differences between the pH levels of the intestinal and fecal samples in trial 2 (Table 3). 

The fecal concentrations of propionic and n-butyric acid were higher in group E2 than P2 (trend for statistical significance in n-butyric acid, *p* = 0.0569; Table 4). The ratios of acetic acid to propionic acid were similar between both diets from trial 2 and also compared to diet E1, but differed markedly from the same ratio in group P1.

In trial 2, post-mortem parameters were obtained. BW did not correlate with body fat content; P2 mice had a significantly higher carcass fat content (*p* < 0.05; Table 5).

Group E2 mice had significantly higher absolute and relative liver weights (*p* < 0.05; Table 5). Correspondingly, the liver GE content was also significantly higher in group E2 (*p* < 0.05). The weight of the GIT was significantly higher in E2 than P2 (*p* < 0.05).

## 4. Discussion

There were deviations in analyzed and labeled nutrient contents that exceeded the legal tolerances (see Table 1) and also marked differences between the batches (P1 vs. P2 and E1 vs. E2). The extruded diets were more similar across batches than the pelleted diets, with P2 differing most from all other diets in starch content. Nutrient levels in natural feed ingredients may differ, so some degree of variance may occur. Analysis of new batches of ingredients before use and consequent re-evaluation of diet formulation is necessary to provide constant nutrient levels in commercial diets. Legal tolerances are not necessarily nutritional tolerances, meaning that they take into account the accuracy of production and analysis, while a greater deviation does not always result in harmful effects for the animal. However, deviations from the labeled nutrient contents are misleading for the researchers using the diet. In laboratory animal diets, standardization is of the utmost importance. The variation of nutrient contents between batches of the same diet (i.e., P1 vs. P2 and E1 vs. E2) does not allow for reproducible experiments on one and the same diet when different batches have to be used.

The content of nitrogen-free extracts alone may be misleading when there is no information on starch and non-starch polysaccharide (NSP) contents. In the diets used in this study, diet P2 had a much lower content of total dietary fiber and contained more starch. The digestibility levels of P2 and E2 did not differ as much as P1 and E1, because the higher total dietary fiber content in E2 most likely counterbalanced the lower starch digestibility in P2. The lower ratio of digestible protein to digestible energy (1.12/1 vs. 1.35/1, respectively) in P2 than E2 explains the higher body fat content of the experimental mice in this study. The combination of the significantly higher body fat content and significantly lower final BW is indicative of marginal protein supply. 

In the present study, the numbers of animals per group were relatively small. For digestibility trials, the number of 6 animals per group is accepted as sufficient [18]. Pair-housing is recommended where possible to minimize stress in social species [19,20], such as the mouse. Considering the 3R principles of reduction, refinement and replacement [20], it is ethically advisable not to use an unnecessarily high number of animals in an experiment. The digestibility data from this trial along with the extremely low standard deviation (see Table 2) proves that the number of animals used is valid for the target parameters. 

As expected, the digestibility of energy in trial 1 and the carbohydrate plus fiber fraction were significantly higher in the extruded diet (E1) compared to the pelleted diet (P1). It can be assumed that lower pre-cecal starch digestibility in P1-fed mice led to higher influx of starch in the large intestine, resulting in higher microbial fermentation in that intestinal site. The significantly lower pH values in the colonic chyme of P1-fed animals and the higher ratio of acetic to propionic acid, which indicate microbial fermentative activity, support that assumption [15,21].

The digestibility of diet E2 was highly similar to E1. However, the digestibility levels for P1 and P2 differed markedly, with P2 being better digestible. This can be explained by the much higher starch content of P2 (43% compared to 27%) and the corresponding lower total dietary fiber content in P2. Pelleted starch is likely to have a higher pre-cecal digestibility than many NSPs [22], and NSPs also negatively influence overall diet digestibility [23].

These differences in starch and NSP content are important with regard to quantitative digestibility. Gelatinized starch is highly digestible, whereas less processed starch is more slowly digested [24]. There is also an interaction between NSPs and starch in the digestive process. NSPs increase chyme viscosity. They may also delay absorption of glucose from starch digestion and alter the intestinal transit time, as well as the microbial fermentation patterns [13,24,25].

Some of the effects postulated above are confirmed by the differences in body composition observed in trial 2. The body fat content measured in trial 2 was significantly higher in P2 than E2, while P2 mice had a significantly lower final BW. This may resemble the “skinny-fat” phenotype [26,27] of individuals that are not obese but have a relatively high body fat content and lower lean body mass. Liver energy content was higher in P2 than E2. Because P2 livers were smaller, it can be concluded that they contained more fat as the most energy-dense compound. The higher body fat and liver fat contents in P2 combined with the lower BW can be indicative of slight protein deficiency. In general, females have small livers during marginal protein supply [28], which seemed to be the case in trial 2. Diet P2 had the lowest protein content of all four diets used in the study and contained much more carbohydrates (Table 1). The protein digestibility of P2 was significantly lower than that of E2 (Table 2). While not resulting in a clinically manifest protein deficiency, this might explain the findings in body composition.

## 5. Conclusions

The results of the present study show that laboratory mouse diets may lack standardization. Firstly, there are differences in composition between batches of the same diet that significantly influence diet digestibility and energy utilization. Secondly, there is an influence of processing on starch gelatinization and digestibility parameters, so that pelleted and extruded diets may not be marketed as identical. Because of the importance of starch content and gelatinization for pre-cecal digestibility and post-ileal microbial fermentation [29], both parameters should be declared on the diet labels.

## Figures and Tables

**Table 1:** Energy and nutrient contents and degrees of starch gelatinization for the diets used in both trials 1 and 2 in the pelleted (P) and extruded (E) diets.

Diet Parameter	Labeled Content	Trial 1		Trial 2	
		P1	E1	P2	E2
Gross energy (MJ/kg)	-	17.23	17.56	16.6	17.3
Dry matter (%)	88.8	89.8	89.8	89.5	88.5
Crude protein (%)	20.6	23.4 *	19.5	18.7	22.5
Crude fat (%)	4.1	3.6	4.3	2.5 *	5.1 *
Crude ash (%)	5.9	5.8	5.6	4.8	6.1
Crude fiber (%)	6.1	6.1	8.2 *	5.2	5.8
Nitrogen-free extracts (%, calculated)	55.0	50.9	52.2	58.3	49.0
Starch (%)	-	27	27	43	28
Starch gelatinization (%)					
- before autoclaving	-	22	64	22	50
- after autoclaving		15	57	17	70
Total dietary fiber (%)	-	27.8	24.5	15.9	23.3
Soluble dietary fiber (%)	-	3.4	4.0	2.0	3.9
Insoluble dietary fiber (%)	-	24.4	20.5	13.9	19.4
Sugars (%)	-	6.2	6.1	4.6	6.5

* Values marked with an asterisk (*) deviate from the labeled value more than the legal tolerance stated in Regulation (EC) No. 767/2009, amended by Regulation (EC) 939/2010. The allowed tolerance for crude protein is ±12.5% for a labeled content between 8 and 24%, and ±1% for crude fat with a labeled content <8%.

**Table 2:** Apparent digestibility of gross energy and nutrients (mean ± SD (%)) in the pelleted (P) and extruded (E) diets from trials 1 and 2.

aD [%]	Trial 1			Trial 2		
	Diet P1 *n* = 11	Diet E1 *n* = 11	*p*	Diet P2 *n* = 16	Diet E2 *n* = 16	*p*
GE	74.8 ± 0.5	80.9 ± 0.6	<0.001	84.4 ± 0.3	81.9 ± 0.6	<0.001
DM	70.8 ± 0.8	77.2 ± 0.7	<0.001	81.3 ± 0.2	77.6 ± 0.8	<0.001
OM	73.7 ± 0.6	80.3 ± 0.5	<0.001	84.7 ± 0.2	80.8 ± 0.7	<0.001
CP	82.2 ± 0.5	81.7 ± 0.9	0.31	83.8 ± 0.3	85.1 ± 0.4	<0.001
EE	91.2 ± 0.7	92.0 ± 0.5	0.09	92.9 ± 1.4	93.4 ± 1.0	0.71
CH+F	69.2 ± 0.7	79.1 ± 0.4	<0.001	85.1 ± 1.7	81.7 ± 4.5	<0.05

aD = apparent digestibility, GE = gross energy, DM = dry matter, OM = organic matter, CP = crude protein, EE = ether extracts, CH+F = carbohydrate + fiber.

**Table 3:** Gastrointestinal and fecal pH values in trials 1 and 2 (mean ± SD).

Sample	Trial 1			Trial 2		
	Diet P1 *n* = 11	Diet E1 *n* = 11	*p*	Diet P2 *n* = 16	Diet E2 *n* = 16	*p*
stomach	3.9 ± 0.5	3.7 ± 0.7	0.46	3.9 ± 0.9	3.6 ± 0.5	0.33
anterior small intestine	6.7 ± 0.3	6.6 ± 0.3	0.54	6.7 ± 0.5	6.7 ± 0.3	0.76
posterior small intestine	7.0 ± 0.4	7.0 ± 0.3	0.70	7.3 ± 0.3	7.2 ± 0.2	0.11
cecum	6.5 ± 1.1	6.9 ± 0.4	0.24	7.2 ± 0.3	7.1 ± 0.2	0.16
colon	6.9 ± 0.3	7.2 ± 0.1	<0.05	7.6 ± 0.3	7.5 ± 0.3	0.14
faeces	7.6 ± 0.1	7.4 ± 0.1	<0.05	7.9 ± 0.2	7.7 ± 0.4	0.33

**Table 4:** Fecal concentrations of organic acids (mean ± SD (mmol/L)) and the ratios of fecal acetic acid to propionic acid for both trials.

mmol/L	Trial 1			Trial 2		
	Diet P1 *n* = 11	Diet E1 *n* = 11	*p*	Diet P2 *n* = 16	Diet E2 *n* = 16	*p*
acetic acid	8.37 ± 1.52	6.95 ± 1.61	0.09	6.44 ± 1.16	6.73 ± 0.97	0.60
propionic acid	1.16 ± 0.15	1.17 ± 0.15	0.92	1.19 ± 0.17	1.25 ± 0.18	0.47
n-butyric acid	1.18 ± 0.21	1.09 ± 0.02	0.57	0.47 ± 0.08	0.55 ± 0.08	0.06
acetic acid/propionic acid	7.15 / 1	5.92/1		5.41/1	5.38/1	

**Table 5:** Results of the carcass analysis from trial 2 (mean ± SD (g)). BW = body weight; GIT = gastrointestinal tract.

Parameter	Diet P2 *n* = 16	Diet E2 *n* = 16	*p*
Gross energy (MJ/kg DM)	25.1 ± 1.9	23.7 ± 1.2	<0.05
Crude protein (% DM)	46.1 ± 7.3	51.9 ± 4.7	<0.05
Fat (% DM)	35.4 ± 9.6	28.4 ± 5.9	<0.05
Ash (% DM)	10.9 ± 1.6	12.1 ± 1.3	<0.05
Liver energy content (MJ/kg)	7.1 ± 0.3	6.9 ± 0.2	0.26
Liver weight in % final BW	4.7 ± 0.6	5.2 ± 0.3	<0.01
GIT weight in % final BW	13.1 ± 1.6	13.8 ± 3.1	<0.05

## Data Availability

All relevant data is listed in the manuscript. Additional information can be requested from the authors upon reasonable request.
